# Measuring mentalizing in youth: further validation of the reflective functioning questionnaire for youth (RFQY-13)

**DOI:** 10.3389/fpsyg.2023.1260281

**Published:** 2023-10-16

**Authors:** Gabriel Martin-Gagnon, Peter Fonagy, Michaël Bégin, Lina Normandin, Karin Ensink

**Affiliations:** ^1^École de Psychologie, Université Laval, Québec, QC, Canada; ^2^Psychoanalysis Unit, Research Department of Clinical, Educational and Health Psychology, University College London, London, United Kingdom; ^3^Département de Psychologie, Université de Sherbrooke, Sherbrooke, QC, Canada

**Keywords:** mentalizing, RF, adolescent, BPD, anxiety, depression, assessment

## Abstract

**Objective:**

This study investigated the psychometric properties, including the factor structure, validity, and reliability of the 13-item Reflective Function Questionnaire for Youth (RFQY-13), using a new scoring system.

**Method:**

A community sample of 414 adolescents and a clinical sample of 83 adolescents (aged 12–21) completed the RFQY, the Borderline Personality Features Scale for Children (BPFS-C), the Beck Youth Inventories (BYI), the Child Behavior Checklist-Youth Self Report (CBCL-YSR) and the Movie for the Assessment of Social Cognition (MASC).

**Results:**

Using the new scoring system, our results demonstrated configural and metric invariance, as well as adequate reliability and validity across both samples for the two-factor structure of the RFQY. The Uncertainty subscale also showed strong associations with psychopathology.

**Discussion:**

The findings show that the RFQY-13, when used with the new coding system, has good psychometric properties and is a reliable measure of mentalizing for adolescents and young adults. We discuss clinical implications, limitations and future directions.

## Introduction

Mentalizing, for the purpose of research often referred to as reflective functioning (RF), denotes the imaginative faculty to perceive and interpret one’s own and others’ mental states within the framework of attachment relationships (Fonagy et al., 2002). While robust RF is tied to enhanced emotional regulation and resilience, deficits in RF are implicated in the emergence of psychopathology among children, adults, and adolescents (Sharp et al., 2016; Ensink et al., 2017; Fonagy et al., 2017; Duval et al., 2018b; Luyten et al., 2020).

In recent times, researchers have zeroed in on the adolescent developmental phase, where RF is integrally linked to self and identity development, as well as the progression of psychopathologies such as Borderline Personality Disorder (BPD; Duval et al., 2018b; Sharp and Rossouw, 2019; Luyten et al., 2021; Martin-Gagnon et al., 2023). Preliminary evidence further associates adolescent RF with depression (Belvederi Murri et al., 2016; Fischer-Kern and Tmej, 2019), anxiety symptoms (Chevalier et al., 2023), and internalizing and externalizing difficulties (Duval et al., 2018a; Seyed Mousavi et al., 2021). Consequently, the enhancement and restoration of mentalizing have emerged as the principal objectives of evidence-based psychotherapies such as Mentalization-Based Treatment (Bateman and Fonagy, 2008; Rossouw and Fonagy, 2012).

Considering the pivotal role of mentalizing in adolescent interventions, the development of reliable measures for assessing RF in adolescents has become a matter of urgency. Although several measures exist, there is a lack of consensus regarding their validity and the key constructs they encapsulate. Different versions of the Reflective Function Questionnaire for Youth (RFQY) have been the subject of validation studies (Ha et al., 2013; Badoud et al., 2015; Fonagy et al., 2016; Duval et al., 2018a; Lund et al., 2022). However, the validity of some of these versions, particularly their scoring procedure and the use of double scored items, has been called into question (Spitzer et al., 2021; Müller et al., 2022). This study seeks to probe the validity of the RFQY-13 in a diverse sample of adolescents from both clinical and community settings.

### Measuring mentalizing: reflective functioning

Over the past few decades, various RF measures, encompassing interviews, experimental tasks, and questionnaires, have been formulated (for a review, see Luyten et al., 2019). While a range of adult RF measures exist, corresponding tools for the youth population require refinement. Among the most frequently employed tools for evaluating adolescents’ RF are the Child and Adolescent Reflective Function Scale (CARFS; Ensink et al., 2014), which is derived from the Child Attachment Interview (Target et al., 2003). Nevertheless, the implementation of these interviews necessitates extensive training, time, and resources (Hill et al., 2007).

To mitigate these challenges, researchers have devised experimental tasks such as the Movie for the Assessment of Social Cognition (MASC; Dziobek et al., 2006) and questionnaires like the Reflective Function Questionnaire (RFQ; Fonagy and Ghinai, 2008). Originally developed for adults, the RFQ was later adapted for adolescents by Sharp et al. (2009) and rebranded as the Reflective Function Questionnaire for Youth (RFQY). Due to the ongoing refinement of the RFQ and RFQY, and the resulting validation studies, numerous versions of the instrument have emerged (Ha et al., 2013; Fonagy et al., 2016; Duval et al., 2018a; Spitzer et al., 2021).

During the initial validation of the RFQ (Fonagy et al., 2016), the authors uncovered an eight-item, two-factor solution, representing (1) Uncertainty (RFQ-U) and (2) Certainty (RFQ-C) about mental states. The authors employed a recoding procedure for the seven-point Likert scale items (1 = do not agree at all, 7 = agree completely) as follows: 0,0,0,0,1,2,3 for RFQ-U and 3,2,1,0,0,0,0 for RFQ-C. For instance, the questionnaire respondent may choose a value of (3) on the seven-point Likert scale item of the RFQ-U subscale. This answer will then go through the recoding procedure in which the initial answer (3) will become a 0. The RFQ-8 also includes double-scored items; although respondents are presented with eight items, four of these are utilized in both subscales but with distinct scoring. The RFQ-8 demonstrated satisfactory fit, validity, reliability, and factorial invariance across adult populations, both clinical and community-based. Moreover, the RFQ-8 has been validated and employed with adolescents in different languages, including French (Badoud et al., 2015), Persian (Seyed Mousavi et al., 2021), and Italian (Bizzi et al., 2022).

It is a common presumption among researchers that high Uncertainty and Certainty scores on the RFQ-8 represent hypomentalizing and hypermentalizing (HMZ), respectively (Badoud et al., 2015; Fonagy et al., 2016; Lund et al., 2022). HMZ is defined as the inference of mental states that lack sufficient supportive evidence (Sharp et al., 2011, 2016). Conversely, hypomentalizing is a diminished mentalizing capacity that impedes the understanding or contemplation of intricate mental states (Fonagy et al., 2016). Though this supposition is theoretically plausible, empirical validation of this assumption has been sparingly undertaken (Duval et al., 2018a).

Despite the widespread usage and validation of RFQ-8, recent criticisms have emerged concerning its application (Spitzer et al., 2021; Müller et al., 2022). Indeed, as Müller et al. (2022) noted, the presence of double-scored items can engender psychometric issues by violating the assumption of uncorrelated residuals. Further, given that respondents can only answer each item once, the double-scoring procedure induces dependence between the subscales. The authors also observed that seven of the eight items on the RFQ-8, which focus on self-RF and the behavioral manifestations of poor RF, are structured to assess Uncertainty. In addition, they contended that due to its item formulation, the RFQ-8 is incapable of reliably measuring HMZ/Certainty and should instead be regarded as a unifactorial tool. Given that HMZ and hypomentalizing are distinct constructs correlated with specific psychopathologies (Sharp et al., 2016; Luyten et al., 2020), a unifactorial instrument undermines the clinical utility of the RFQ-8.

### Reflective functioning questionnaire for youth

The Reflective Function Questionnaire for Youth (RFQY) was initially devised with 46 items employing a six-point Likert scale (1 = strongly disagree, 6 = strongly agree) and was subdivided into two subscales (Scale A and B) with distinct scoring methods. Scale A used a median scoring approach where extreme scores signified poor RF, and medium scores signified optimal RF. Conversely, Scale B employed a Likert scale, where higher scores corresponded to better RF. The total score of the RFQY was derived from the summation of Scales A and B. The English version of the RFQY was first validated by Ha et al. (2013) on a sample of 146 inpatient adolescents. The preliminary validation of the RFQY-46 was conducted using the instrument’s total score, revealing commendable construct validity and reliability.

However, when Duval et al. (2018a) endeavored to explore the factor structure of the RFQY-46 using a sample of French Canadian adolescents from the community (*n* = 533), they failed to substantiate the initial bifactor structure. They addressed the problem with the initial scoring system by introducing a Likert scale without any recoding procedure. Employing the Likert scale, they identified a three-factor solution delineating Uncertainty/Confusion, Interest/Curiosity, and Certainty about mental states. This revised version contained 25 items and delivered superior reliability coefficients compared to the initial study by Ha et al. (2013).

Following these developments, Lund et al. (2022) validated a 13-item version of the RFQY in Danish, mimicking the scoring scheme (0,0,0,0,1,2) and bifactor structure of the RFQ-8 in a community sample (*n* = 644), a clinical but non-BPD sample (*n* = 64), and a BPD sample (*n* = 181) of adolescents aged 14–18 (total *n* = 889). Through exploratory factor analysis, the authors pared down the original 46 items to 13, bifurcated into two factors: Uncertainty (RFQY-U) and Certainty (RFQY-C) about mental states. The RFQY-13 exhibited a good model fit, satisfactory internal consistency, and significant correlations with BPD features. The RFQY also demonstrated discriminant validity, with RFQY-U mean scores being significantly lower in the community sample than in the other groups. For the RFQY-C, the community sample had a significantly higher mean score than the BPD group, but no difference was observed with the non-BPD group.

In summation, a myriad of RFQY versions have been validated to assess adolescent mentalizing. Previous studies have raised scoring and validity concerns with the RFQ-8 and RFQ-46 (Duval et al., 2018a; Spitzer et al., 2021; Müller et al., 2022). The RFQY-13 (Lund et al., 2022) presents promising results with fewer items, facilitating the assessment of RF impairments and circumventing the double scoring procedure. Nonetheless, further validation is warranted.

### This study

This study is indeed highly necessary to extend the understanding of the psychometric properties of the RFQY-13. The use of the Likert scale coding from Duval et al. (2018a) is an effective way to address the identified issues with previous coding systems, as pointed out by Müller et al. (2022).

The objectives of this study are well-structured, aiming to assess various aspects of the RFQY-13. Firstly, the verification of the bifactor structure (Uncertainty and Certainty) as proposed by Fonagy et al. (2016) and Lund et al. (2022) in both clinical and community samples of adolescents will provide robustness to the validity of the RFQY-13. This invariance testing across different types of samples is critical for establishing the measure’s applicability in diverse contexts.

Secondly, examine the discriminant validity of the RFQY-13 to ensure its effectiveness in differentiating between community and clinical samples. The expectation that clinical participants will report higher RFQY-U scores, in line with previous research, seems appropriate and provides a clear hypothesis to be tested.

Lastly, the test of convergent validity is a key aspect of this study, particularly the investigation of the associations of the RFQY-13 with BPD features, anxiety, depression, internalizing and externalizing difficulties, hypomentalizing, and HMZ. The hypothesis that the RFQY-13, particularly the Uncertainty subscale, will be strongly associated with BPD features, anxiety, depression, internalizing and externalizing difficulties for both groups is indeed in line with previous findings. The theoretical alignment of Uncertainty and Certainty with hypomentalizing and HMZ respectively, and the expected positive correlation between them, also provide solid ground for a meaningful interpretation of the results.

## Method

### Participants and procedure

Our study engaged a total of 497 participants drawn from both community settings and clinical mental health services. Approval for the research was granted by Laval University’s ethics committee in Canada. Parental consent was secured for participants aged 12–13, in accordance with ethical guidelines. For adolescents aged 14 and above, we solicited and received consent directly from the participants themselves, in compliance with Article 21 of the provincial regulations governing consent to participation in research. The inclusion criteria specified an age range of 12 to 21 and proficiency in French.

Participants were apprised of the research and invited to participate, and they completed a series of questionnaires via a unique identifier link leading to the Qualtrics online platform. The community-based cohort (*N* = 414) was sourced from school and university environments. The age of these participants ranged from 12 to 21 years (*M* = 15.05, SD = 1.45), with females making up the majority (62%). The ethnic distribution was primarily Caucasian (91.1%), followed by Black American (2.2%), Asian (1.7%), Hispanic (1%), Middle Eastern (0.7%), Native American (0.2%), and other (1.9%).

The clinical cohort (*N* = 83) was drawn from an outpatient psychiatric program and a university psychology clinic. The age of these participants also ranged from 12 to 21 years (*M* = 16.63, SD = 2.41), with a larger percentage of females (74%). The ethnic distribution here was primarily Caucasian (85.7%), followed by other (9.1%), Hispanic (2.6%), Asian (1.3%), and Middle Eastern (1.3%).

### Measures

The Reflective Function Questionnaire for Youth (RFQY; Sharp et al., 2009; Duval et al., 2018a; Lund et al., 2022) is a self-report questionnaire to assess RF in adolescents. The RFQY-13 consists of 13 items divided into two subscales reflecting Uncertainty (7 items) and Certainty (6 items) about mental states. We used the Likert scale coding from Duval et al. (2018a) in which a mean score is calculated for each scale without any recoding procedure. Each item uses a six-point Likert scale ranging from 1 (strongly disagree) to 6 (strongly agree). This questionnaire has been validated with clinical (Ha et al., 2013) and community (Duval et al., 2018a; Lund et al., 2022) samples.

The Movie for the Assessment of Social Cognition (MASC; Dziobek et al., 2006; Bossé-Chartier, 2013) is a computerized video task developed to assess social cognition and mentalization abilities. Participants watch a 15-min video portraying four characters getting together for a dinner party. The video pauses 45 times to ask the participant about a character’s mental state (e.g., “What is Betty’s intention?”). Each question consists of four possible responses which reflect (1) HMZ, (2) hypomentalizing, (3) no mentalizing, and (4) good mentalizing. Subscales’ total scores are calculated by adding their frequencies. The MASC is considered an ecologically reliable tool for assessing mentalizing and social cognition in adolescents (Ha et al., 2013; Fossati et al., 2017).

The Borderline Personality Features Scale for Children (BPFS-C; Crick et al., 2005; Ensink et al., 2020) is a self-report questionnaire assessing BPD features in minors. This questionnaire was adapted from the BPD subscale of the Personality Assessment Inventory (PAI; Morey, 1991). BPFS-C consists of 24 items divided into four subscales of six items each. The scales are (1) affective instability, (2) identity issues, (3) negative relationships (4) self-harm. On a 5-point Likert scale, items range from 1 (not true at all) to 5 (always true). A total score is obtained by adding the scores of the four subscales. According to Chang et al. (2011), a total score of 66 represents the optimal cutoff score for discriminating BPD in adolescents.

The Beck Depression Inventory for Youth (BDI-Y; Beck et al., 2005). Depression was measured using the Depression subscale of the Beck Inventory for Youth (BYI; Beck et al., 2005). This widely used and well-validated self-report questionnaire measures depression symptoms, including youth’s negative emotions, thoughts and sleep disturbances. Each item uses a 4 points Likert scale ranging from 0 (never) to 3 (always). The 20 response scores are added to calculate the total score, then transformed into T scores according to the participant’s gender and age. Scores of ≥70 are considered extremely elevated, 60–69 are moderately elevated, 55–59 are mildly elevated, and < 55 are average.

The Beck Anxiety Inventory for Youth (BAI-Y; Beck et al., 2005) is a self-report questionnaire from the BYI measuring physiological symptoms of anxiety, worries and fears regarding the future, loss of control, and reaction to peers and school performance. Like the depression inventory, this questionnaire has 20 items and uses four-point Likert scales ranging from 0 (never) to 3 (always). The BAI-Y score interpretation is identical to the BDI-Y.

The Child Behavior Checklist - Youth Self Report (CBCL-YSR; Achenbach and Rescorla, 2001) is a 112-item questionnaire assessing child and adolescent emotional and behavioral functioning, namely internalizing and externalizing behaviors. Each item is rated on a three-point Likert scale and divided into eight subscales. By summing up the individual scores of the relevant subscales, externalizing and internalizing scores can be calculated; withdrawal, somatic complaints, anxiety, and depression comprise the internalizing scale, whereas social problems, aggressive behavior, and delinquent behavior comprise the externalizing scale. Scores of ≥69 are considered clinically significant, 64–69 suggest some difficulties and < 64 are not significant.

### Data analysis

The factor structure of the RFQY-13 was evaluated using a (multiple group) Confirmatory Factor Analysis (CFA) model with raw data and maximum likelihood estimation in Mplus 8.5 (Muthen and Muthén, 2017). In alignment with the guidelines laid out by Brown (2015), a CFA was initially performed separately on each group, which was then followed by a series of multigroup CFAs (carried out on both groups simultaneously). We began with an equal form model (configural), then progressed to a model with fixed factor loadings (metric), and finally to a model with both fixed factor loadings and fixed intercepts (scalar).

The model’s fit was evaluated using several fit indices: Comparative Fit Index (CFI), Tucker-Lewis Index (TLI), root mean square error of approximation (RMSEA) with a 90% confidence interval, standardized root mean square (SRMR), the chi-square (χ^2^), and the chi-square to degrees of freedom ratio (χ^2^/df). These indices denote an acceptable fit of the model when: CFI ≥ 0.90, TLI ≥ 0.90, RMSEA ≤0.06, SRMR ≤0.08 (or close to these values), χ^2^/df ≤ 3 and when the χ^2^ is not significant (Hu and Bentler, 1999; Schumacker and Lomax, 2004). The nested model comparisons were evaluated using the difference between three fit statistics (Δχ^2^, ΔCFI, ΔRMSEA). Following Chen’s (2007) guidelines, we used a criterion of −0.01 change in CFI paired with a change of 0.015 in RMSEA to compare the configural and metric models as well as metric and Scalar models.

Additionally, internal consistency, correlations, and group comparisons were carried out using the Statistical Package for the Social Sciences (SPSS 28; IBM). We estimated the internal consistency of the subscales using Macdonald’s ω coefficients (McDonald, 1999). The ω is more reliable and less restrictive than Cronbach’s alpha while providing the same interpretation guidelines (Brown, 2015; Trizano-Hermosilla and Alvarado, 2016).

For testing the discriminative validity of the instrument between clinical and community participants, assuming scalar invariance has been achieved to enable meaningful comparison of the group means, independent t-tests will be used. To test the convergent validity of the RFQY with other constructs, Pearson correlation analyses were employed.

## Results

### Demographics and descriptive statistics

Descriptive statistics, including means, standard deviations and group comparison for both community and clinical groups, are presented in Table 1.

**Table 1:** Means, standard deviations, *p*-values and effect size (Cohen *d*’s) for the community and clinical groups.

	Clinical		Community			
Variables	*M*	*SD*	*M*	*SD*	*t*	*d*
RFQY-U	3.76	0.95	3.20	1.01	4.61**	0.56
RFQY-C	3.37	0.85	3.46	0.89	−0.80	−0.10
BPFS-C	63.15	17.50	55.24	14.31	4.16**	0.52
BDI-Y	62.32	11.37	49.89	10.16	8.20**	1.18
BAI-Y	60.71	12.44	49.45	10.28	7.43**	1.04
CBCL-INT	67.55	9.83	55.26	11.11	8.10**	1.13
CBCL-EXT	55.28	8.05	51.09	8.86	3.41**	0.48
MASC Hyper	8.90	3.53	8.12	2.98	1.62	0.25
MASC Hypo	10.12	4.43	7.25	2.79	4.75**	0.83

RFQY, Reflective Function Questionnaire for Youth; U, RFQY Uncertainty scale; C, RFQY Certainty scale; BPFS-C, Borderline Personality Features Scale for Children; BDI-Y, Beck Depression Inventory for Youth; BAI-Y, Beck Anxiety Inventory for Youth; M, mean; SD, standard deviations. **p < 0.001, *p < 0.05.

**Figure 1:**
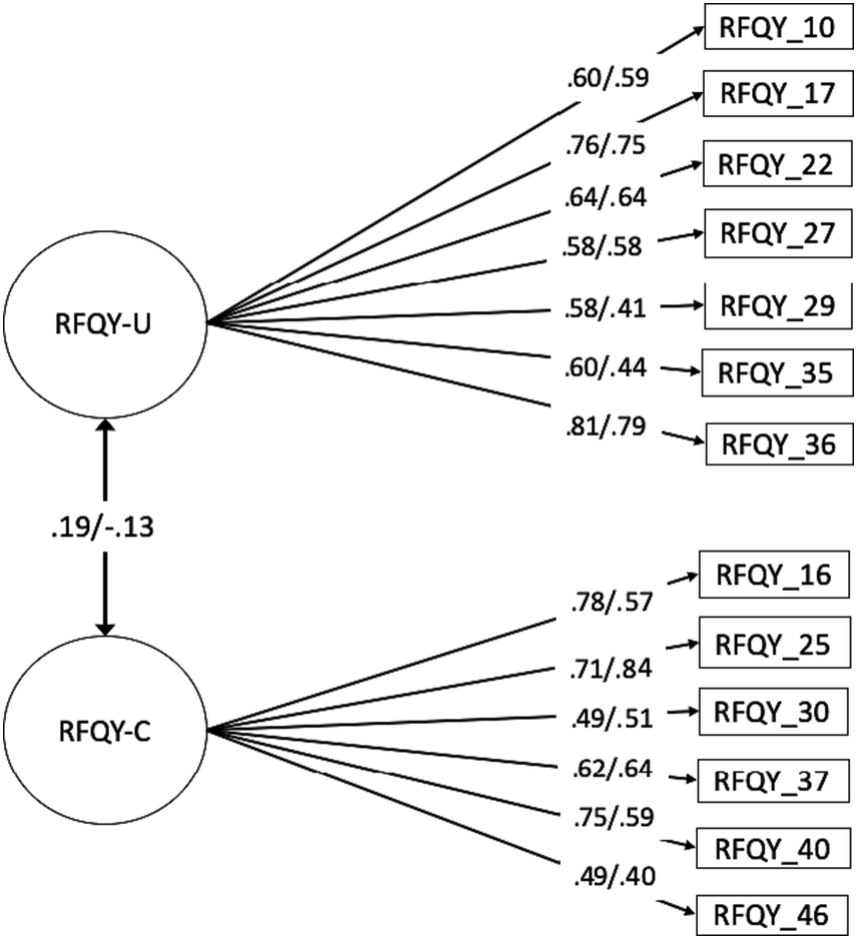
Confirmatory factor analysis for the community **(left)** and clinical **(right)** groups.

The Confirmatory Factor Analysis (CFA) was performed using both subscales of the RFQY-13 for the clinical and the community sample separately. The CFA model yielded favorable fit indices for the community sample: χ^2^(59) = 96.914, *p* = 0.001, χ^2^/df = 1.340, CFI = 0.981, TLI = 0.975, RMSEA = 0.037, SRMR = 0.037; as well as for the clinical sample: χ^2^(57) = 74.194, *p* = 0.062, χ^2^/df = 1.301, CFI = 0.941, TLI = 0.919, RMSEA = 0.060, SRMR = 0.075. Importantly, all items displayed significant factor loadings (λ ≥ 0.40) for both groups, as shown in Figure 1.

Following this, measurement invariance was tested through a sequence of multigroup CFAs incorporating: (1) equal form, (2) equal factor loadings, and (3) equal factor loadings and intercepts. The multigroup CFA with equal forms (configural) exhibited a good fit for the combined samples: χ^2^(114) = 193.759, *p* = < 0.001, χ^2^/df = 1.699, CFI = 0.963, TLI = 0.949, RMSEA = 0.053, SRMR = 0.049, suggesting structural invariance. Additionally, imposing a constraint on factor loadings (metric model) resulted in a good fit: χ^2^(125) = 205.388, *p* = < 0.001, χ^2^/df = 1.643, CFI = 0.962, TLI = 0.953, RMSEA = 0.051, SRMR = 0.053. This model demonstrated metric invariance when compared to the configural model: Δ χ^2^(11) = 11.630, *p* = 0.392, ΔCFI = 0.001, ΔRMSEA = 0.002. Furthermore, imposing a constraint on factor loadings and intercepts (scalar model) resulted in an acceptable fit: χ^2^(136) = 250.326, *p* = < 0.001, χ^2^/df = 1.840, CFI = 0.946, TLI = 0.938, RMSEA = 0.058, SRMR = 0.061. However, the scalar model did not yield an improved fit compared to the metric model: Δ χ^2^(11) = 44.937, *p* = <0.001, ΔCFI = 0.016, ΔRMSEA = 0.007.

Internal consistency estimates for the community sample were moderate for the RFQY-U subscale (ω = 0.847) and the RFQY-C subscale (ω = 0.811). The clinical sample had moderate estimates for the RFQY-U subscale (ω = 0.785) and acceptable for the RFQY-C subscale (ω = 0.746). Estimates for the combined sample were acceptable for both the RFQY-U subscale (ω = 0.842) and the RFQY-C subscale (ω = 0.800).

Finally, we used gender and age as grouping variables to test the instrument’s invariance for the community sample.

Comparing girls and boys, the configural model exhibited a good fit: χ^2^(114) = 207.844, *p* = <0.001, χ^2^/df = 1.699, CFI = 0.946, TLI = 0.926, RMSEA = 0.066, SRMR = 0.054, suggesting structural invariance. Additionally, the metric model resulted in a good fit: χ^2^(125) = 218.739, *p* = <0.001, χ^2^/df = 1.749, CFI = 0.946, TLI = 0.932, RMSEA = 0.063, SRMR = 0.061. This model demonstrated metric invariance when compared to the configural model: Δ χ^2^(11) = 10.895, *p* = 0.452, ΔCFI = 0.000, ΔRMSEA = 0.003. Furthermore, the scalar model showed an acceptable fit: χ^2^(136) = 259.830, p = < 0.001, χ^2^/df = 1.910, CFI = 0.928, TLI = 0.918, RMSEA = 0.069, SRMR = 0.067 but did not yield an improved fit compared to the metric model: Δ χ^2^(11) = 44.937, *p* = <0.001, ΔCFI = 0.016, ΔRMSEA = 0.007.

Regarding age, when comparing adolescents aged 12–15 with adolescents aged 16–21 the configural multigroup CFA exhibited a good fit: χ^2^(114) = 205.202, *p* = <0.001, χ^2^/df = 1.699, CFI = 0.951, TLI = 0.933, RMSEA = 0.063, SRMR = 0.056, suggesting structural invariance. Additionally, the metric model resulted in a good fit: χ^2^(125) = 214.142, *p* = <0.001, χ^2^/df = 1.749, CFI = 0.952, TLI = 0.940, RMSEA = 0.060, SRMR = 0.060. This model demonstrated metric invariance when compared to the configural model: Δ χ^2^(11) = 8.939, *p* = 0.627, ΔCFI = 0.002, ΔRMSEA = 0.003. Furthermore, the scalar model resulted in an acceptable fit: χ^2^(136) = 239.324, *p* = <0.001, χ^2^/df = 1.910, CFI = 0.945, TLI = 0.936, RMSEA = 0.062, SRMR = 0.064 and showed scalar invariance compared to the metric model: Δ χ^2^(11) = 25.182, *p* = 0.008, ΔCFI = 0.007, ΔRMSEA = 0.004.

### Discriminant validity

Table 1 presents the group mean comparisons between the community and clinical groups for all variables under consideration. However, as previously stated, while testing the invariance of the RFQY-13 between community and clinical groups, scalar invariance was not achieved. As a result, we did not proceed with interpreting group comparisons.

### Convergent validity

Bivariate correlations between the RFQY subscales and the main study variables are presented in Table 2.

**Table 2:** Bivariate correlations between key study variables for the community (left) and clinical (right) groups, respectively.

	1	2	3	4	5	6	7	8	9
1. RFQY-U	—								
2. RFQY-C	0.15**/−0.17	—							
3. BPFS-C	0.75**/0.54**	0.17**/−0.28**	—						
4. BDI	0.57**/0.37**	0.19**/−0.27*	0.72**/0.48**	—					
5. BAI	0.48**/0.30**	0.18**/−0.23*	0.68**/0.45**	0.82**/0.73**	—				
6. INT	0.53**/0.31**	0.16**/.-19	0.69**/0.39**	0.64**/0.47*	0.68**/0.56**	—			
7. EXT	0.53**/0.36**	0.23**/−0.18	0.63**/0.45**	0.39**/0.19	0.31**/0.17	0.51**/0.22*	—		
8. MASC Hyper	−0.09/0.04	−0.04/−0.01	−0.03/0.01	−0.02/0.09	−0.03/0.07	0.06/−0.07	−0.06/0.29*	—	
9. MASC Hypo	0.01/−0.03	−0.34**/0.03	−0.03/−0.02	−0.06/0.06	0.10/0.28*	−0.07/0.26*	−0.13/0.17	0.17/0.44**	—

RFQY, Reflective Function Questionnaire for Youth; U, RFQY Uncertainty subscale; C, RFQY Certainty subscale; BPFS-C, Borderline Personality Features Scale for Children; BDI, Beck Depression Inventory for Youth; BAI-Y, Beck Anxiety Inventory for Youth; INT, Internalizing difficulties; EXT, externalizing difficulties; MASC Hyper, Hyper-mentalizing subscale; MASC Hypo, hypomentalizing subscale. **p < 0.01, *p < 0.05.

Both samples manifested significant positive correlations between the RFQY-U and all psychopathology measures. The RFQY-C exhibited a positive correlation with all psychopathology measures and a negative correlation with the MASC hypomentalizing scale in the community group. In contrast, within the clinical group, the RFQ-C showed negative correlations with BPD features, depression, and anxiety, and displayed marginally non-significant associations with internalizing (*p* = 0.06) and externalizing difficulties (*p* = 0.08). Furthermore, the RFQY-U and RFQY-C subscales demonstrated a positive association within the community group, while they indicated a negative, albeit marginally non-significant (*p* = 0.06), association within the clinical group.

## Discussion

This study aimed to explore the psychometric properties of the RFQY-13 in a sample of adolescents from both clinical and community settings, utilizing Duval et al.’s (2018a) scoring method. The initial objective was to validate the bifactor structure as identified by Lund et al. (2022), as well as to assess the measure’s invariance across clinical and community samples of adolescents. We successfully replicated the bifactor structure, characterized by Uncertainty and Certainty about mental states. The CFAs revealed an adequate fit of this structure and impressive factor loadings for both individual and combined samples. Moreover, our findings affirmed the measure’s configural and metric invariance, implying that the RFQY-13 items maintain the same factor structure and equivalent factor loadings across community and clinical samples. Furthermore, the internal consistency of the subscales was found to be acceptable in both samples, exhibiting similar Omega coefficients to those reported by Lund et al. (2022). In the community sample, the RFQY-13 showed configural and metric invariance when comparing boys and girls, as well as scalar invariance when comparing younger and older adolescents in the community.

In addressing the psychometric concerns raised by Müller et al. (2022) regarding the RFQ-8, our findings suggest that the RFQY-13, employing Duval’s Likert scale scoring method, is a reliable instrument. In prior iterations, the RFQ-8 and RFQY-13 employed a rescoring procedure associated with certain psychometric issues, leading to a reduction in the nuance and variance of participant responses (Müller et al., 2022). However, our results indicate that the use of the RFQY-13 with Likert scale scoring, while eliminating the problematic rescoring procedure, avoids these complications while demonstrating exceptional validity and reliability. Furthermore, this approach simplifies the scoring process and application of the instrument by researchers and clinicians.

### Discriminant validity

The second objective of this study was to assess the discriminant validity of the RFQY-13, specifically its capability to differentiate between community and clinical adolescent groups. However, scalar invariance was not achieved while comparing the RFQY-13 community and clinical groups. This means that latent means could not be compared meaningfully.

### Convergent validity

The third objective was to assess the convergent validity of the RFQY-13 in relation to BPD features, anxiety, depression, internalizing and externalizing difficulties, hypomentalizing, and HMZ. As anticipated, RFQY-U scores exhibited strong associations with all psychopathology measures in both the community and clinical samples. This suggests that Uncertainty about mental states represents a transdiagnostic risk factor underpinning psychological distress and difficulties in adolescents. Our study holds an advantage over prior research in that we incorporated measurements of a wide array of psychological difficulties. This enabled us to demonstrate significant associations between Uncertainty, BPD features, internalizing and externalizing difficulties, as well as anxiety and depression, in both community and clinical groups within a single study. The results align with previous research revealing strong associations between adolescents’ Uncertainty about mental states and BPD features (Duval et al., 2018a,b; Vahidi et al., 2021; Lund et al., 2022; Martin-Gagnon et al., 2023), internalizing and externalizing difficulties (Badoud et al., 2016; Seyed Mousavi et al., 2021), depression (Li et al., 2020), and anxiety (Martin-Gagnon et al., 2023). Moreover, our findings are consistent with evidence suggesting that Uncertainty is a central mentalizing deficit associated with psychological difficulties in adults (Fonagy et al., 2016; Euler et al., 2021; Müller et al., 2022).

One unexpected finding in our study was the differential association of the RFQY-C subscale with psychopathology in the community and clinical samples. Specifically, RFQY-C was found to be positively correlated with psychopathology in the community sample, but negatively correlated in the clinical sample. This finding is somewhat challenging to interpret.

RFQY-C, which focuses on cognitive mentalizing of others (with statements such as “I know exactly what my close friends are thinking”), captures the notion of excessive Certainty. This reflects a belief of complete understanding of others’ thoughts, feelings, and intentions, indicative of poor mentalizing and potentially leading to negative interpersonal consequences. Fonagy et al. (2016) proposed that this excessive Certainty about others’ mental states might serve as an adaptive mechanism in response to developmental weaknesses in mentalizing capabilities. Optimal mentalizing acknowledges the inherent uncertainty and limitations in understanding others’ mental states.

The function of Certainty may diverge between high-pathology and low-pathology groups. For adolescents in the clinical group, higher levels of Certainty may serve as a protective mechanism against psychological distress and disorganization. Conversely, in the community sample, higher levels of Certainty might be linked with increased psychological difficulties due to its negative interpersonal repercussions.

In our study, we utilized both the RFQY and MASC, which provided us with an opportunity to explore the relationships between the RFQY scales and HMZ and hypomentalizing, as measured by the MASC. However, contrary to our expectations, we did not observe an association between the RFQY-U and the MASC hypomentalizing subscale, nor between the RFQY-C and the MASC HMZ subscale. This suggests that the RFQY Uncertainty and Certainty scales are measuring distinct constructs that are unrelated to HMZ and hypomentalizing. These results lend further support to Müller et al. (2022) conclusion that the RFQ does not measure HMZ, given that the HMZ subscale of the MASC is currently viewed as a reference measure of HMZ (McLaren et al., 2022). Additionally, we observed a correlation between MASC hypomentalizing and measures of anxiety and internalizing difficulties in the clinical sample, suggesting that hypomentalizing represents an additional mentalizing deficit that is clinically relevant.

Given our findings, it’s clear that the RFQY-U scale is not measuring Hypomentalizing, leading us to consider what it does capture. An examination of the RFQY-U items suggests that it predominantly gauges self and affective mentalizing, as well as instances of mentalizing failures in the context of emotional dysregulation or impulsivity. Items such as “I often get confused about what I am feeling” and “I do not always know why I do what I do,” along with “strong feelings often cloud my thinking,” underscore this interpretation. Based on this content and the overlap with the Uncertainty/Confusion subscale in Duval et al.’s (2018a) RFQY-25, we posit that the RFQ-U scale measures Uncertainty and Confusion about mental states. We suggest that the explicit naming of this subscale could facilitate the interpretation of research findings and the application of these results in adolescent clinical practice.

In summary, our study results indicate that the RFQY-13 offers a succinct measure of mentalizing for adolescents and young adults, showing valid psychometric properties. The confirmed two-factor structure comprises (1) Uncertainty and (2) Certainty about mental states. Additionally, Uncertainty/Confusion about mental states was found to be strongly associated with psychological difficulties and distress in young individuals, underscoring its clinical relevance for identifying mentalizing difficulties and informing interventions. In our study, RFQY Uncertainty and RFQY Certainty emerged as distinct constructs, separate from the MASC scales of HMZ and Hypomentalizing. This suggests that the RFQY-13 and MASC’s HMZ and Hypomentalizing scales can be viewed as complementary tools, each capturing different facets of mentalizing impairments.

Although the MASC is frequently employed in research, its application in clinical settings is more challenging, given that it’s a task requiring at least 15 min, compared to the shorter questionnaire format of the RFQY-13. This raises the question of whether items capturing HMZ and Hypomentalizing can be developed and incorporated into the RFQY to make it a more comprehensive tool for mentalizing assessment.

This study also has limitations that need to be taken into account. First, while the sample size of the community group was substantial (*n* = 414), the clinical group was comparatively smaller (*n* = 83). As such, further validation of the RFQY-13 with larger clinical populations is necessary to confirm the scale’s utility across different contexts. Future research should also aim to further probe the associations between the RFQY-13 subscales and various psychopathology measures, particularly the Certainty subscale, to better understand its predictive utility in clinical settings.

Second, although we used a range of psychopathology measures in our study, we did not administer assessments to determine specific psychiatric diagnoses among participants. Therefore, future research employing clinical samples and focusing on distinct disorders is warranted. Such investigations would help determine whether particular mentalizing difficulties are associated with specific diagnoses or if these difficulties are ubiquitous across a range of psychiatric disorders. This will further enhance our understanding of the potential role and implications of mentalizing difficulties in psychopathology.

Third, the RFQY-13 did not show scalar invariance, preventing us from comparing latent means between clinical and community samples. This may be viewed as a statistical constraint, but it is highly improbable that mentalizing levels would possess equivalent intercepts across adolescents from populations as diverse as clinical and community groups (Fonagy et al., 2016).

The study’s findings have several important implications for research and clinical assessment of mentalizing in adolescents, as well as for informing clinical interventions. The improved psychometric properties and simplified scoring procedure of the RFQY-13 mean that clinicians and researchers can now use the measure with more confidence. The measure is brief which has definite advantages in terms of efficiency when assessing mentalizing deficits and should make it possible for mentalizing to be assessed widely in research and clinical contexts. The RFQY-13 may also be useful for clinicians by alerting them to mentalizing difficulties characterized by Uncertainty/confusion or excessive certainty that can be addressed clinically. As such it draws attention to mentalizing difficulties that are not widely focused on and could facilitate the development of interventions for these difficulties.

## Conclusion

In conclusion, our study provides valuable insights into the RFQY-13’s psychometric properties and its potential application in assessing mentalizing in adolescents and young adults. However further research is necessary to address the study’s limitations and expand our understanding of the practical implications in clinical contexts of the RFQY-13.

## Data availability statement

The raw data supporting the conclusions of this article will be made available by the authors, without undue reservation.

## Ethics statement

The studies involving humans were approved by Comités d’éthique de la recherche avec des êtres humains de l’Université Laval. The studies were conducted in accordance with the local legislation and institutional requirements. Written informed consent for participation in this study was provided by the participants’ legal guardians/next of kin.

## Author contributions

GM-G: Conceptualization, Formal analysis, Investigation, Methodology, Writing – original draft, Writing – review & editing. PF: Writing – review & editing. MB: Conceptualization, Writing – review & editing. LN: Data curation, Investigation, Methodology, Project administration, Writing – review & editing. KE: Conceptualization, Methodology, Supervision, Writing – review & editing.
